# Pleiotropic regulatory genes *bldA*, *adpA* and *absB* are implicated in production of phosphoglycolipid antibiotic moenomycin

**DOI:** 10.1098/rsob.130121

**Published:** 2013-10

**Authors:** Roman Makitrynskyy, Bohdan Ostash, Olga Tsypik, Yuriy Rebets, Emma Doud, Timothy Meredith, Andriy Luzhetskyy, Andreas Bechthold, Suzanne Walker, Victor Fedorenko

**Affiliations:** 1 Department of Genetics and Biotechnology, Ivan Franko National University of Lviv, Hrushevskoho st. 4, Lviv 79005, Ukraine; 2 Albert-Ludwigs-University of Freiburg, Pharmazeutische Biologie, Stefan-Meier-Strasse 19, 79104 Freiburg, Germany; 3 Department of Microbiology and Immunobiology, Harvard Medical School, 4 Blackfan Circle, Boston, MA 02115, USA; 4 Helmholtz Institute for Pharmaceutical Research, Saarland Campus, Building C2.3, 66123 Saarbrucken, Germany

**Keywords:** pleiotropic regulators, *Streptomyces*, moenomycin A, *adpA*, *bldA*, *absB*

## Abstract

Unlike the majority of actinomycete secondary metabolic pathways, the biosynthesis of peptidoglycan glycosyltransferase inhibitor moenomycin in *Streptomyces ghanaensis* does not involve any cluster-situated regulators (CSRs). This raises questions about the regulatory signals that initiate and sustain moenomycin production. We now show that three pleiotropic regulatory genes for *Streptomyces* morphogenesis and antibiotic production—*bldA*, *adpA* and *absB*—exert multi-layered control over moenomycin biosynthesis in native and heterologous producers. The *bldA* gene for tRNA^Leu^_UAA_ is required for the translation of rare UUA codons within two key moenomycin biosynthetic genes (*moe*), *moeO5* and *moeE5*. It also indirectly influences moenomycin production by controlling the translation of the UUA-containing *adpA* and, probably, other as-yet-unknown repressor gene(s). AdpA binds key *moe* promoters and activates them. Furthermore, AdpA interacts with the *bldA* promoter, thus impacting translation of *bldA*-dependent mRNAs—that of *adpA* and several *moe* genes. Both *adpA* expression and moenomycin production are increased in an *absB-*deficient background, most probably because AbsB normally limits *adpA* mRNA abundance through ribonucleolytic cleavage. Our work highlights an underappreciated strategy for secondary metabolism regulation, in which the interaction between structural genes and pleiotropic regulators is *not* mediated by CSRs. This strategy might be relevant for a growing number of CSR-free gene clusters unearthed during actinomycete genome mining.

## 2. Introduction

Moenomycins (Mms) are a small family of secondary metabolites of actinomycete origin that display a number of remarkable traits in terms of their chemistry and biology [1]. Classified as phosphoglycolipids, they result from a unique assembly of glycoside- and isoprene-derived moieties bridged by 3-phosphoglyceric acid—an unprecedented building block in secondary metabolism (SM). Moenomycins strongly interfere with the growth of mainly Gram-positive bacteria, including VRE and MRSA pathogens, through direct inhibition of peptidoglycan glycosyltransferases (PGTs). High potency of these antibiotics and their unique mode of action explain much of the industrial and academic interest in them. We have recently identified genes for moenomycin production (*moe* gene cluster) by *Streptomyces ghanaensis* ATCC14672 and harnessed them for generation of a number of useful phosphoglycolipid analogues [2]. However, moenomycin production by either *S. ghanaensis* or heterologous hosts must be significantly increased before combinatorial biosynthesis can be a reliable source of novel moenomycins for biological tests or chemical modifications. We therefore set out to explore the regulation of moenomycin production by *S. ghanaensis*, with the ultimate goal of using the gained knowledge for strain improvement.

In the vast majority of studied cases, the transcriptional regulators of actinomycete SM gene clusters form a two-tiered network, with genes for cluster-situated regulators (CSRs) and global (or pleiotropic) regulators scattered over the genome and unlinked to SM pathways [3,4]. Global regulators affect the expression of more than one SM pathway by modulating the expression of CSR genes. SM pathways often have more than a single associated CSR, in which case one of the CRSs is an ultimate regulator of antibiotic production (responsible for activation of structural antibiotic biosynthesis genes), while others may act either singularly, on the ultimate regulatory gene, or pleiotropically, on unrelated and unlinked genes. It should be emphasized that ‘topology-based’ classification of regulators (cluster-situated versus global) does not predict function. That is, a CSR gene may encode any of the following: (i) an ultimate regulator; (ii) a true pleiotropic regulator [5] or ultimate regulator with ‘cross-talk’ properties [6]; or (iii) a regulator of a distal gene cluster [7]. As one of the hallmarks of actinomycete SM gene clusters, CSRs have attracted the interest of researchers, particularly as a tool to develop antibiotic overproducers, and they are often considered an essential layer of transcriptional control over secondary metabolite production [8].

In contrast to the model described above, moenomycin biosynthesis does not involve CSRs [9]. No CSR genes are found in the *moe* cluster; the presence of essential moenomycin-specific regulatory gene(s) elsewhere in the *S. ghanaensis* genome is unlikely given that we were able to recreate moenomycin production in several heterologous hosts [10]. Although CSR-free SM gene clusters in actinomycetes have been considered the exception rather than the rule [11,12], the number has increased steadily as numerous whole genomes have been sequenced and analysed [13–16]. These gene clusters represent a poorly understood archetype of regulation of actinomycete SM, where CSRs are not involved. *In silico* analysis of *moe* genes revealed the presence of TTA leucine codons in two key *moe* genes, *moeO5* and *moeE5*. TTA is the rarest codon in actinobacteria [17] and, in streptomycetes, it is generally found in genes with auxiliary functions (SM, aerial mycelium and spore formation, cryptic). In *Streptomyces coelicolor*, mature tRNA^Leu^_UAA_ (specified by *bldA* gene) is only formed during late stationary growth, defining the onset of hyphae and antibiotic production [18,19]. *BldA* regulation occurs via the presence of UUA codons within CSR genes [20]. Recent work on ipomicin biosynthesis has provided initial evidence that *bldA* also regulates the translation of structural SM genes [21]. We hypothesize that *bldA* regulates moenomycin production at the level of translation of mRNA of the key structural *moe* genes. However, it is unlikely that *bldA* is the only regulator of moenomycin production given the importance of transcriptional control over SM (*vide supra*). Indeed, our previous *moe* promoter titration studies pointed to the existence of transcriptional activator(s) of *moe* gene expression [10]. In this study, we show that AdpA_gh_, an *S. ghanaensis* orthologue of well-known *S. coelicolor* and *Streptomyces griseus* master regulator AdpA [22–24], is an important and direct activator of *moe* gene expression. The translation of UUA-containing *adpA_gh_* mRNA is dependent on *bldA*-encoded tRNA, although this dependence is not absolute. Finally, we provide circumstantial evidence that AdpA_gh_ expression is regulated at the posttranscriptional level through the action of the *absB_gh_* gene, encoding an orthologue of *S. coelicolor* RNase III [25]. Together these data outline the involvement of three interacting global regulatory genes, *absB*–*adpA*–*bldA*, in control of a CSR-free secondary metabolic pathway. The first gene, *absB*, directly regulates *adpA* expression, *bldA* regulates the translation of both *adpA* and moenomycin structural genes and *adpA* directly influences moenomycin production. The regulatory influence of these genes on moenomycin production is effective in *S. ghanaensis* as well as several heterologous hosts. Our data and data from recent literature allow us to propose that AdpA and BldA may constitute a central regulatory component relevant to many SM pathways lacking cluster-situated, pathway-specific regulatory genes.

## 3. Results

### 3.1. *In silico* analysis of *Streptomyces ghanaensis* genome suggests the involvement of AdpA in moenomycin production

**Figure 1.**
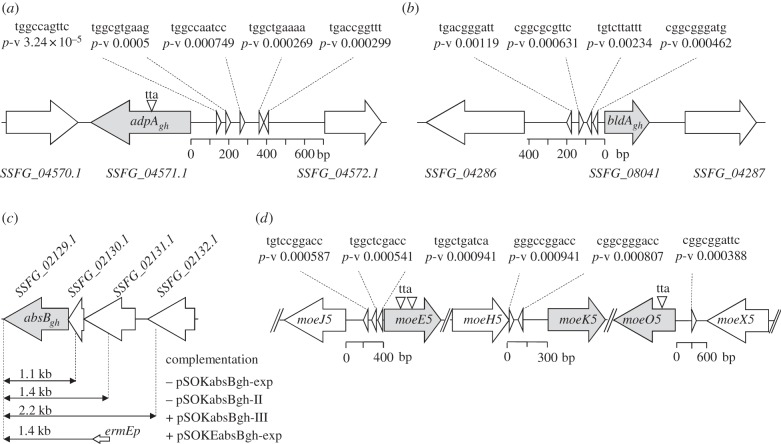
Fragments of *S. ghanaensis* genome relevant to this study. Triangles indicate position of AdpA-binding sites as predicted *in silico*. Respective score values are given near the binding sites. (*a*) The *adpA_gh_*-containing region with the adjacent genes. The distance between start and stop codons is shown. (*b*) Gene *bldA_gh_* with its promoter region. The putative start of mature tRNA is shown. (*c*) Operon containing *absB_gh_* and constructs used for complementation of *S. ghanaensis *Δ*absB_gh_* mutant. (*d*) Positions of high-scoring AdpA_gh_-binding sites within intergenic regions of *moe* cluster 1 studied in this work. The distance between start and stop codons is shown.

Recent studies portrayed the transcription factor AdpA as one of the most versatile regulators of *Streptomyces* biology [24,26–29], including the expression of CSR-free secondary metabolic gene clusters [16]. In *S. coelicolor* and *S. griseus,* AdpA is known to influence other regulators, such as tRNA^Leu^_UAA_ (BldA) and RNaseIII (AbsB). The latter regulates AdpA abundance via ribonucleolytic cleavage of its mRNA. As the moenomycin biosynthetic cluster does not contain any specific regulatory genes, it is an excellent test bed to investigate the possibility of combined SM regulation from AdpA, AbsB and BldA. Our laboratory previously identified an orthologue of *absB* in *S. ghanaensis* [10]. The *absB-*containing chromosomal regions of *S. coelicolor* and *S. ghanaensis* are syntenous. Presumably, *absB_gh_* belongs to the transcriptional unit which comprises three genes: *SSFG_02131.1*, *SSFG_02130.1* and *SSFG_02129.1* (*absB_gh_*) (figure 1).

In our *in silico* analysis [10] of *S. ghanaensis*, we identified an AdpA orthologue in *S. ghanaensis* and designated it as *adpA_gh_*. The coding sequence of *adpA_gh_* contains one TTA codon (figure 1), at the same position as other *adpA_gh_* orthologues [23,30–32]. Genes for several AdpA_gh_ paralogues are present in the *S. ghanaensis* genome (see the electronic supplementary material, table S1). Additionally, a single copy of the tRNA^Leu^_UAA_ gene was identified in the *S. ghanaensis* genome (designated as *bldA_gh_*; figure 1).

We mined the promoter regions of *adpA_gh_, bldA_gh_, absB_gh_* and *moe* clusters for the presence of AdpA operator sequences [33]. As expected, such sequences were revealed within *adpA_gh_p* and *bldA_gh_p* (figure 1). AdpA operator-like sites were identified within many intergenic regions of the *moe* cluster 1 (data not shown). Particularly, promoter regions of the key genes *moeE5, moeK5* and *moeO5*, responsible for production of the earliest monosaccharide MmA intermediate [2], contain three, two and one such sites, respectively (figure 1). The presence of an AdpA orthologue in the *S. ghanaensis* genome and its respective operator sequences within the *moe* cluster indicated that it may have a role in the regulation of moenomycin production.

### 3.2. Moenomycin production is completely abolished in *Streptomyces ghanaensis adpA* and *bldA* mutants, and increased in the *absB* mutant

**Figure 2.**
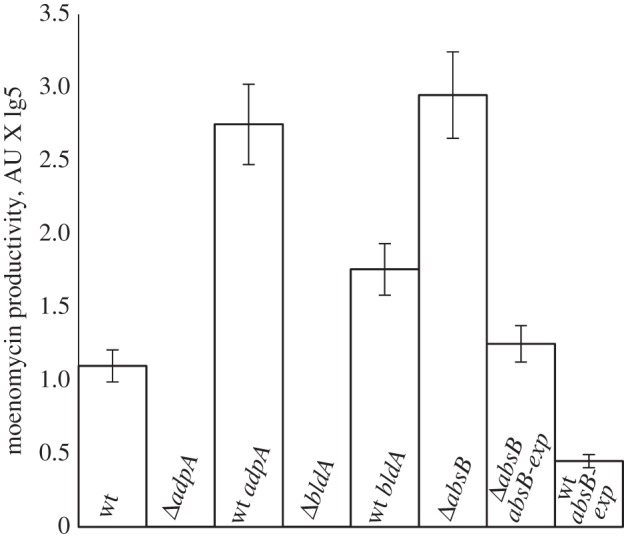
Levels of moenomycin production by various *S. ghanaensis* strains. Column labels: wt, *Δ*adpA—wild-type and *adpA_gh_* null mutant, respectively; wt adpA—wild-type strain overexpressing *adpA_gh_*; *Δ*bldA—*bldA_gh_*-minus mutant; wt bldA—wild-type strain overexpressing *bldA_gh_*; *Δ*absB—*absB_gh_*-minus mutant; *Δ*absB absB-exp—*absB_gh_*-minus mutant expressing plasmid for complementation pSOKEabsBgh-exp; wt absB-exp—wild-type strain overexpressing *absB_gh_*.

**Figure 3.**
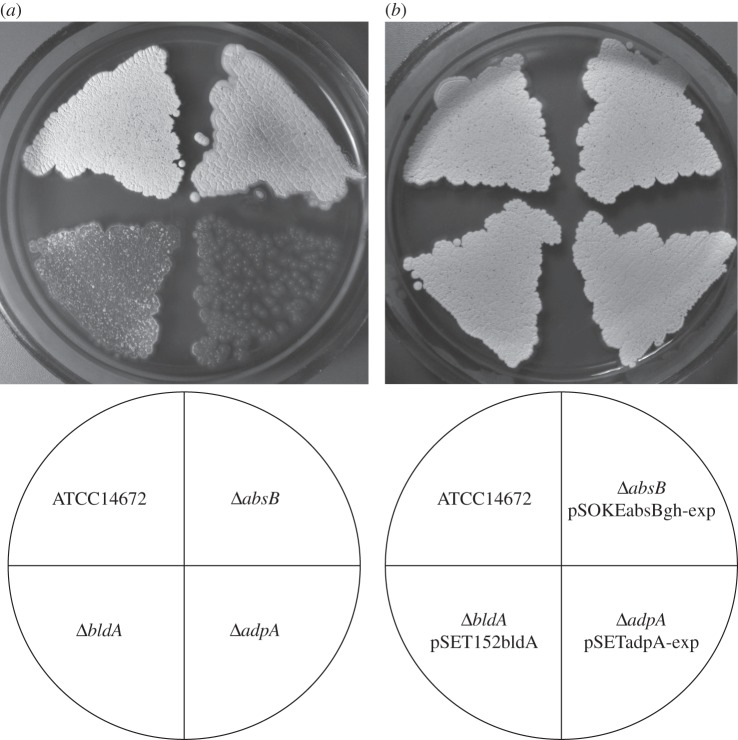
Lawns of *S. ghanaensis* mutants described in this study. (*a*) Wild-type *S. ghanaensis* ATCC14672, *S. ghanaensis* *ΔabsB_gh_*, *S. ghanaensis* *ΔbldA_gh_* and *S. ghanaensis* *ΔadpA_gh_* (clockwise from the top left) were grown on TSB agar medium. (*b*) Respective mutants carrying plasmids for complementation, pSOKEabsBgh-exp, pSET152bldA and pSETadpA-exp.

Deletion of *adpA_gh_* in the *S. ghanaensis* chromosome completely abolished moenomycin production, as determined by LC-MS (figure 2) and bioassays. No mass peaks corresponding to the earliest known moenomycin precursors [2] were found in the extracts of *adpA_gh_* mutant (*ΔadpA_gh_*), showing that moenomycin production was blocked at the initial first steps. Knockout of *adpA_gh_* had a significant influence on the morphological development *S. ghanaensis*. On solid media, a phenotype of *S. ghanaensis* *ΔadpA_gh_* resembled that of the ‘bald’ (*bld*) mutants described for streptomycetes (figure 3 and [34]). AdpA proteins in other species are key developmental regulators, and their deletion has been reported to lead to substantial morphological defects [26,32,35].

The moenomycin production and morphology in the *ΔadpA_gh_* were restored to the wild-type state upon introduction of an intact copy of *adpA_gh_* (plasmid pSETadpA-exp). Insertion of an extra copy of *adpA_gh_,* under the control of a strong constitutive promoter *ermEp* (plasmid pTESadpA-exp), caused a 2.5-fold increase in moenomycin production compared with the control strain (figure 2).

Like the *ΔadpA_gh_*, *S. ghanaensis* *ΔbldA_gh_* did not produce MmA or any of its intermediates (figure 2). Deletion of *bldA_gh_* impaired morphological development of *S. ghanaensis* (figure 3); in particular, aerial mycelium formation was considerably delayed compared with the wild-type strain (figure 3).

The introduction of a native copy of *bldA_gh_* into *ΔbldA_gh_* (plasmid pSETbldA) restored the moenomycin production and normal timing of morphogenesis, implying that the *ΔbldA_gh_* phenotype is solely due to the absence of tRNA^Leu^_UAA_. The introduction of a second copy of *bldA* (pSET152bldA) into the wild-type strain led to a slight (1.6-fold on average) but reproducible increase in moenomycin production (figure 2).

**Figure 4.**
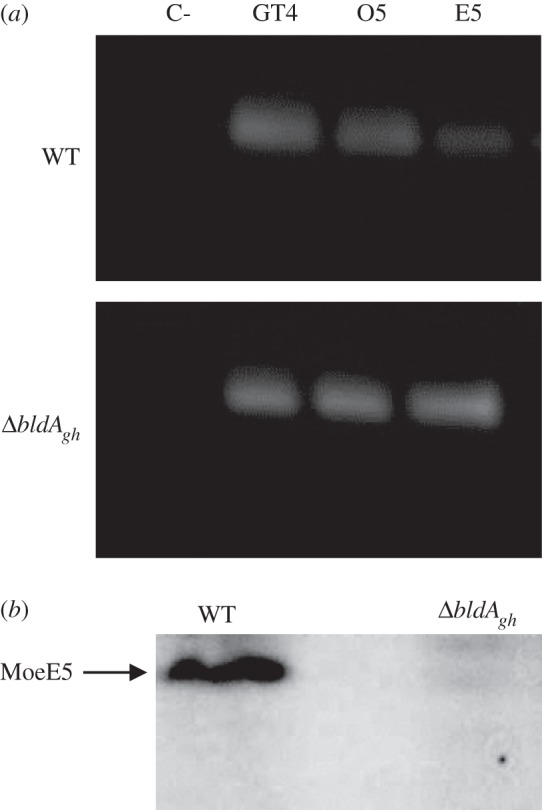
The *bldA_gh_* gene directly affects translation of *moeE5*. (*a*) RT-PCR analysis of *moeE5*, *moeO5 and moeGT4* transcription in *S. ghanaensis* wild-type (WT) and *bldA*-deficient (*Δ*bldA_gh_) strains. Lane C-, negative control (*rrnA* amplification from RNA sample in absence of RT). (*b*) Western blot analysis of cell-free lysates from WT and *ΔbldA*_*gh*_ strains. The lysates were obtained from mycelium harvested in moenomycin production phase (TSB, 72 h) and probed with anti-MoeE5 rabbit serum (raised as described in §5).

The transcription and translation of several *moe* genes was analysed in further detail to determine whether the *bldA* mutation affected moenomycin production directly (by blocking the translation of UUA-containing *moeO5* and *moeE5* mRNAs) or indirectly (by arresting *adpA_gh_* expression). Semiquantitative RT-PCR analysis of *moeO5*, *moeE5* and *moeGT4* showed that their transcription was not decreased in *ΔbldA_gh_*; in fact, it appeared to be increased (figure 4). Western blots revealed that MoeE5 protein is present in the cell-free lysate of the wild-type strain, but not in that of *ΔbldA_gh_* (figure 4), indicating a direct regulatory influence on the expression of TTA-containing *moe* genes by tRNA^Leu^_UAA_.

The RNase III-deficient mutant (*ΔabsB_gh_*) produced on average 2.7 times more moenomycin compared with the parental strain (figure 2). On solid media, *ΔabsB_gh_* differed subtly from the wild-type (figure 3). Chromatograms of the methanol extracts from the three aforementioned mutants and the wild-type demonstrated little qualitative difference beyond the moenomycin-related peaks (see the electronic supplementary material, figure S1). Nevertheless, new mass peaks seemed to occur in both *ΔbldA_gh_* and *ΔadpA_gh_*, and one peak disappeared in *ΔadpA_gh_* extracts; detailed characterization of these peaks was not pursued.

Bioinformatic analysis indicated that *absB_gh_* and two upstream genes (*SSFG_02131.1* and *SSFG_02130.1*) are separated by 2 and 19 bp, indicative of transcriptional operon organization (figure 1). For complementation of *S. ghanaensis* *ΔabsB_gh_*, a series of integrative plasmids with different portions of this putative operon were constructed (for details, see §5). Only the plasmid containing *absB_gh_* in *cis* with the two upstream genes (pSOKabsBgh-III; figure 1) decreased moenomycin production to the wild-type level, suggesting that the *absB_gh_* is the last gene in a tricistronic message. Additional complementation experiments were designed to confirm that *absB_gh_* alone is sufficient to restore the wild-type phenotype. *absB_gh_* under the control of *ermEp* (pSOKEabsBgh-exp) was integrated into the *S. ghanaensis* *ΔabsB_gh_* chromosome, and the resulting strain produced 2.5 times less moenomycin than the wild-type strain. Introduction of the same plasmid (pSOKEabsBgh-exp) into the wild-type strain resulted in significantly decreased antibiotic biosynthesis (figure 2).

### 3.3. GusA reporter systems reveal the interactions of regulators with *moe* genes and each other

**Figure 5.**
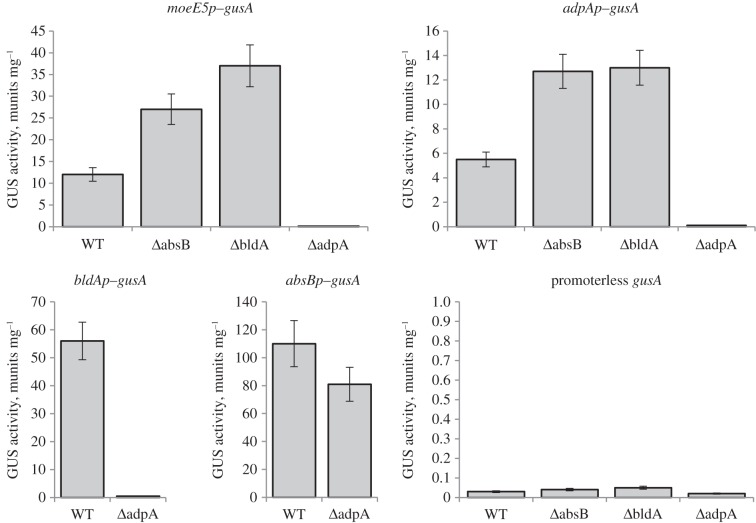
Transcriptional activity of selected promoters in *S. ghanaensis *Δ*absB*, *ΔbldA* and *ΔadpA* strains. WT, *Δ*absB, *Δ*bldA and *Δ*adpA correspond to wild-type, *absB_gh_*, *bldA_gh_* and *adpA_gh_* null mutant strains, respectively, of *S. ghanaensis* expressing *gusA* from different promoters. The *moeE5p*, *adpAp*, *absBp* and *bldAp* correspond to promoters of *moeE5*, *adpA_gh_*, *absB_gh_* and *bldA_gh_*, respectively.

**Figure 6.**
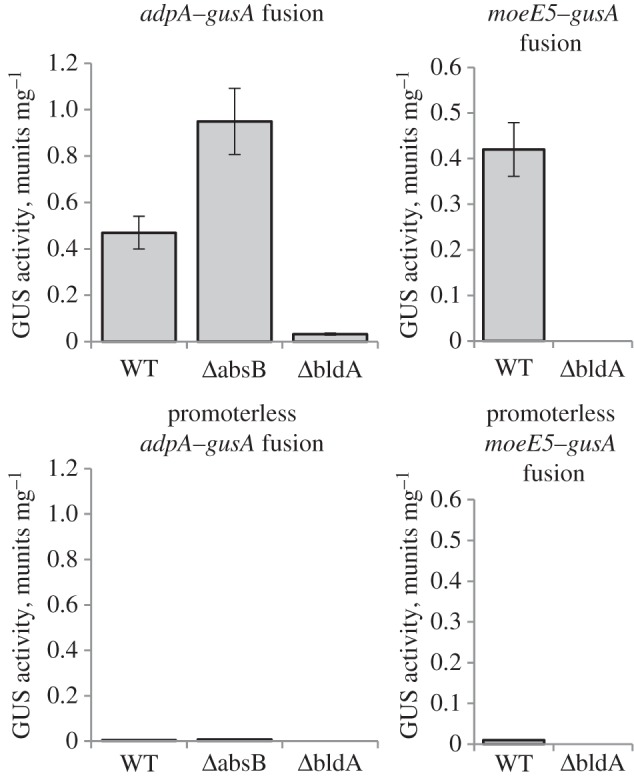
Translation of AdpA_gh_ and MoeE5 is strongly affected on *bldA_gh_*-minus background. WT, *Δ*absB and *Δ*bldA correspond to wild-type, *absB_gh_* and *bldA_gh_* null mutant strains, respectively, of *S. ghanaensis* expressing *gusA* fused to tested genes along with their promoters. *adpA* and *moeE5* correspond to genes *adpA_gh_* and *moeE5*, respectively. As a negative control, promoterless versions of the above genes were fused to *gusA* and introduced into respective strains; these constructs had marginal or no GusA activity.

The recently described β-glucuronidase (GusA) reporter system [36] was applied to investigate the functional connection between the aforementioned pleiotropic regulators and *moe* genes. First, we measured transcription from the promoter of key structural gene *moeE5* (*moeE5p*) in all of the *S. ghanaensis* mutants. The wild-type strain had relatively high levels of transcription from *moeE5p* (see, for comparison, the activity of other SM gene promoters [36]), but we failed to detect transcription in the *ΔadpA_gh_* strain (figure 5). The *moeE5* transcription was increased more than twofold and threefold from wild-type levels in *S. ghanaensis* *ΔabsB_gh_* and *ΔbldA_gh_* strains, respectively (figure 5), in agreement with RT-PCR data (figure 4). While the pattern of *moeEp* activity in *ΔadpA_gh_* and *ΔabsB_gh_* is as anticipated [25], increased levels of *moeE5p* transcript in the *ΔbldA_gh_* are somewhat unexpected. A plausible explanation is that *moeE5p* might be a target of an as-yet-unknown repressor(s) positively regulated by BldA, in which case the deletion of *bldA* would remove the repressive signal. To further delineate the involvement of *bldA_gh_* in the translational regulation of moenomycin production, we analysed GusA activity of translational fusions of *gusA* to *moeE5* (plasmid pmoeE5transl) and *adpA_gh_* in a *ΔbldA_gh_* background. We found no GusA activity in *ΔbldA_gh_* carrying *moeE5–gusA* fusion (figure 6), underscoring the essentiality of *bldA_gh_* for translation of the two UUA codons in *moeE5* mRNA. Surprisingly, GusA activity was detected in the *ΔbldA_gh_* strain carrying *adpA_gh_–gusA* fusion, although it was much weaker (15-fold) than that in wild-type strain (figure 6). This observation can be attributed to mistranslation of *adpA_gh_* UUA codon in the absence of tRNA^Leu^_UAA_ [37,38]. As the expression of AdpA in other cases has been shown to be strictly dependent on BldA [23,32,39], our data set a precedent for this important group of pleiotropic activators.

Next, we analysed *adpA_gh_p* transcription. In comparison to the wild-type strain, *adpA_gh_p* levels (figure 5) increased 2.3-fold in *S. ghanaensis* *ΔabsB_gh_* and were almost undetectable in the *ΔadpA_gh_* strain (figure 5). We also measured the level of translation when *adpA_gh_p* and the entire *adpA_gh_* genes were fused to *gusA* (padpAtransl; see §5) and found it in *ΔabsB_gh_* to be double that of wild-type (figure 6). Thus, AdpA_gh_ acts as a positive activator of its own expression and its activity is increased in the absence of ribonucleolytic activity of AbsB_gh_. This conclusion is supported by observations in other streptomycetes [25,40]. Similar to our *moeE5p* data, *adpA_gh_p* activity was also significantly increased in the *ΔbldA_gh_* strain (figure 5), suggesting the existence of an unidentified *bldA*-dependent repressor(s) of AdpA_gh_-regulated promoters.

There was no difference between *absB_gh_p* transcriptional activity in *ΔadpA_gh_* and wild-type strains, indicating that AdpA_gh_ does not influence the transcription of *absB_gh_*.

At the same time, we revealed almost complete cessation of *bldA_gh_* transcription in the *ΔadpA_gh_* strain (figure 5).

### 3.4. Adpa_gh_ interacts with promoters of *bldA_gh_*, *adpA_gh_* and key *moe* genes

**Figure 7.**
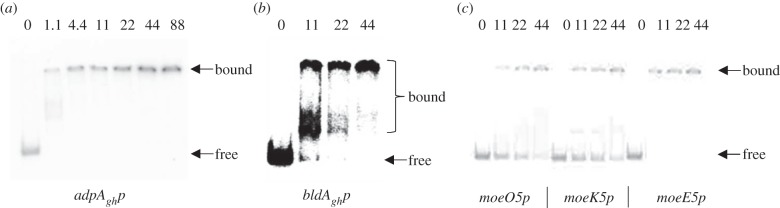
AdpA_gh_ interacts with promoter of its own gene (*a*), *bldA_gh_* (*b*) and several *moe* genes (*c*). EMSA showing binding of purified AdpA_gh_ to various promoter regions comprising *in silico*-predicted AdpA-binding sites. *moeO5p, moeK5p, moeE5p, adpA_gh_p* and *bldA_gh_p* correspond to promoter regions of *moeO5, moeK5, moeE5, adpA_gh_* and *bldA_gh_* genes, respectively. Bands corresponding to protein–DNA complexes (bound) and free DNA (free) are indicated. The final amount of AdpA_gh_ (pmol) is indicated above each line.

The GusA reporter data suggested that AdpA_gh_ is a transcriptional activator that regulates its own expression as well as that of *bldA_gh_* and *moe* genes. To test this, we set out to demonstrate AdpA_gh_ binding to *moeO5, moeK5, moeE5*, *bldA_gh_* and *adpA_gh_* promoter regions using electrophoretic mobility shift assay (EMSA). A C-terminally His_6_-tagged derivative of AdpA_gh_ was overexpressed in *Escherichia coli* and purified to homogeneity (see the electronic supplementary material, figure S2). Increasing amounts of AdpA_gh_-His were incubated with radiolabelled DNA probes corresponding to the promoter regions of interest, and the complexes were separated by native gel electrophoresis. Purified AdpA_gh_-His was bound to the promoter regions of *moeO5, moeK5, moeE5*, *bldA_gh_* and *adpA_gh_* in quantities as low as 1.1–11.0 pmol. Increasing concentrations of AdpA_gh_ resulted in more than one protein–DNA complex for *moeO5p* and *moeK5p* (figure 7), in agreement with multiple AdpA-binding sites predicted for these promoters *in silico*. While bioinformatics analysis predicted three putative AdpA-binding sites within *moeE5p*, only one shifted band was presented in the case of *moeE5p*. Unlabelled *adpA_gh_* promoter competed with the radiolabelled one for AdpA_gh_ (see the electronic supplementary material, figure S3), while a non-specific DNA fragment (the *SCO3812* promoter region) was not recognized by AdpA_gh_ (see the electronic supplementary material, figure S4).

AdpA_gh_ formed three different *bldA_gh_p*–AdpA_gh_ complexes suggesting the presence of multiple AdpA-binding motifs within *bldA_gh_p*. Increasing concentrations of AdpA_gh_ caused a transition from multiple bands to a single retarded band, suggesting that above a certain AdpA_gh_ concentration, all AdpA operators will be occupied by the recombinant protein. Finally, we confirmed that AdpA_gh_ binds to its own promoter (figure 7). Low concentrations of AdpA_gh_ (4.4 pmol) caused the appearance of intermediate nucleoprotein complexes, whereas saturation of the reaction mixture with AdpA_gh_ resulted in the formation of single band.

### 3.5. Absb, AdpA and BldA are important for moenomycin production by heterologous hosts

Previously, we demonstrated the successful expression of *moe* clusters in different streptomycetes [9,10]. To investigate whether the regulatory network we discovered in *S. ghanaensis* also operates in these heterologous hosts, we analysed the moenomycin production of the strains of *S. coelicolor* and *Streptomyces lividans* impaired in *adpA*, *absB* and *bldA* genes.

**Figure 8.**
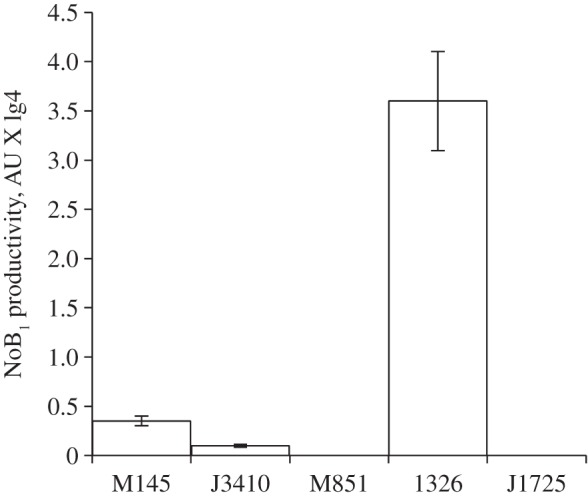
Levels of nosokomycin B_1_ production by various streptomycetes expressing cosmid moeno38-5. Column labels: M145, J3410 and M851—wild-type, *rnc* (*absB*)-minus and *bldH* (*adpA*)-minus mutants of *S. coelicolor*, respectively; 1326 and J1725—wild-type and *bldA* null mutant of *S. lividans,* respectively.

To determine the level of moenomycins biosynthesis on a *ΔabsB-*background, a cosmid moeno38-5 [10] carrying the main part of *moe* cluster 1 and directing the production of nosokomycin B_2_ (NoB_2_) was introduced into *S. coelicolor* *ΔabsB* strain J3410 [41]*. S. coelicolor* J3410 moeno38-5^+^ was grown in parallel with a control strain *S. coelicolor* M145 moeno38-5^+^ and NoB_2_ was quantified. On average, J3410 moeno38-5^+^ accumulated 20% less biomass than M145 moeno38-5^+^ and produced three times less NoB_2_ compared with the control strain (figure 8). These data correlate with the results of reporter experiments, where we observed a 1.5-fold decrease in *moeE5* transcription in a *ΔabsB_gh_* strain compared with a control M145 strain (data not shown). Our results suggest that the AbsB RNase III-mediated regulatory pathway is important for moenomycin production even in other streptomycete heterologous hosts.

Next, we tested NoB_2_ production in *adpA*-deficient *S. coelicolor* M851 and *bldA*-deficient *S. lividans* J1725 strains. Mutant and parental strains carrying cosmid moeno38-5 did not differ in growth rate, but NoB_2_ production was completely abolished (figure 8). No *moeE5p* activity was revealed in *S. coelicolor* M851. NoB_2_ production was restored to M851 and J1725 upon introduction of *adpA_gh_* and *bldA_gh_*, respectively (data not shown).

## 4. Discussion

The vast majority of natural product biosynthetic gene clusters do contain one or more CSR genes. Expression of the latter is shown in many cases to be dependent on global pleiotropic regulators, for example AdpA [26,42]. Once produced, CSR proteins directly activate the transcription of structural biosynthetic genes [3,4,43]. However, a growing body of data suggest that cluster-situated layers of regulation are not an obligatory component of actinomycete secondary metabolic pathways. The elucidation of the genetic organization of the erythromycin biosynthetic cluster in the early 1990s provided the first evidence of an SM pathway lacking CSRs [11,12,44]. The list of ‘CSR-free’ gene clusters continues to grow; they direct the production of secondary metabolites, as chemically diverse as polyketides (erythromycin), both ribosomal and non-ribosomal peptides (thiostrepton, albonoursin, pacidamycins) [45,46], nucleoside analogues, phosphoglycolipids [1,14,15,47,48] and acarbose-like natural products [49,50].

**Figure 9.**
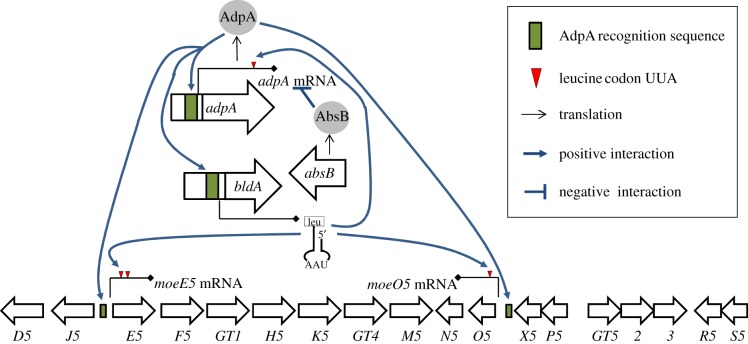
A model of the regulatory pathway that governs moenomycin biosynthesis in *S. ghanaensis*.

It is important to understand whether the expression of different ‘CSR-free’ gene clusters has a common mechanism(s) or principle of regulation. In this study, we show that expression of one such gene cluster, that for moenomycin production, is directly governed by two pleiotropic regulators, one of which is likely to be also under the influence of a third regulator. The described regulatory network is summarized in figure 9. Here, two pleiotropic regulators AdpA and BldA are involved in direct and multi-layered control over moenomycin production, whereas another protein, AbsB, limits AdpA abundance via ribonucleolytic activity. We would like to underscore the reciprocity of functional interactions enabling strict control over moenomycin production. The pleiotropic transcriptional regulator AdpA directly binds to the promoter regions of antibiotic biosynthetic genes as well as its own promoter. BldA contributes to the availability of developmentally regulated tRNA^Leu^_UAA_, the absence of which limits the translation of both *adpA* and *moe* structural genes. Finally, *absB*-encoded RNaseIII influences antibiotic production by modulating AdpA abundance in addition to other, poorly understood way(s) evident from our heterologous expression experiments. This kind of regulatory network was initially elucidated in model streptomycetes, *S. coelicolor* and *S. griseus* [39], where it also governs antibiotic production. However, unlike these model cases, the influence of the studied regulators on moenomycin production does not appear to be mediated by CSRs.

According to available genomic data, *absB*, *adpA* and *bldA* orthologues are omnipresent in *Streptomyces* genomes, providing the necessary foundation for their evolution as a regulatory system that bypasses CSRs. Of the three regulators, BldA directly regulating CSR-free pathways has been extensively studied in other systems [21,47], while the involvement of AdpA was most substantially confirmed in the case of grisemycin biosynthesis [16,51]. The presence of AdpA operator sequences in the promoters of structural genes is another important indication of its role in the regulation of CSR-free pathways. A cursory *in silico* analysis indicates that the gene clusters for the biosynthesis of thiostrepton, pacidamycin, albonoursin, acarbose and puromycin all contain putative AdpA operator sequences within certain intergenic regions. Some of these clusters include structural genes containing TTA codons as well (tunicamycin, albonoursin, erythromycin and puromycin clusters). Echoing the idea put forward by Higo *et al.* [24], we think that the low DNA-binding specificity of AdpA may be the key to the evolution of its control over CSR-free antibiotic biosynthetic gene clusters. In fact, AdpA was shown to form the largest bacterial regulon known to date with over 500 genes under its direct control. AdpA, like no other pleiotropic transcriptional factor of *Streptomyces*, would therefore be capable of putting laterally acquired antibiotic biosynthesis gene clusters under growth phase-dependent control. We note that another moenomycin biosynthetic gene cluster, located within the giant plasmid pSCL4 of *Streptomyces clavuligerus* ATCC27064 [52], may provide complementary evidence for the importance of AdpA control over the secondary metabolome. Despite numerous attempts, we failed to detect the production of moenomycins by *S. clavuligerus* (B. Ostash 2012, unpublished data). The *moe* gene clusters of *S. ghanaensis* and *S. clavuligerus* are syntenous and respective (homologous) gene products share 71–94% similarity [1]; yet, intergenic regions of *S. clavuligerus moe* cluster do not contain AdpA-binding sites (data not shown). It will be interesting to determine the distribution of AdpA operators within silent gene clusters of streptomycetes to further elucidate the role of AdpA in control of the secondary metabolome.

The promoter region of tRNA^Leu^_UAA_-encoding gene *bldA_gh_* is under the transcriptional control of AdpA_gh_. However, *adpA_gh_* contains one UUA codon in the middle of the coding sequence, putting it under BldA_gh_ translational control. In *S. coelicolor*, *S. griseus* and *S. clavuligerus* [23,30,32,39] this control is very strict, while in *S. ghanaensis *Δ*bldA_gh_* AdpA_gh_ is still detected, albeit at a considerably decreased level. AdpA_gh_ mistranslation in the *ΔbldA_gh_* mutant could account for this observation, which, to our knowledge, would be the first case for the AdpA family of proteins. However in the same *S. ghanaensis* *ΔbldA_gh_* strain, we failed to detect epimerase MoeE5, which contains two TTA codons, indicating that mistranslation of TTA codons does not occur 100% of the time in the *bldA_gh_*-minus background.

The severe morphological defects of *S. ghanaensis* *ΔbldA_gh_* vary under different growth conditions, which may contribute to AdpA_gh_ expression. The complexity and conditionality of the *bldA* phenotype is well known in *S. coelicolor* [53,54]. It is the chief reason for ongoing debate as to whether *bldA* constitutes a ‘true regulatory device’ [55,56] or just a ‘wiring’ of the other regulatory networks [57,58]. Our data as well that of Wang *et al*. [21] unequivocally demonstrate that a *bldA* deletion directly abrogates the translation of UUA-containing transcripts and, subsequently, antibiotic production. Hence, *bldA* is a unique tRNA, absence of which indeed creates a regulatory event in the form of infinite delay of the translation of UUA^+^ transcripts. Recent work showed that, compared with the more abundant tRNAs, accumulation of primary *bldA* transcript began at earlier stages, and BldA tRNA scaffold does not determine its regulatory role [56]. Availability of mature BldA may thus be regulated by posttrancriptional modification, but no evidence for that is available. Function of BldA is likely to be more conditional than that of transcriptonal factors, which might be manifested in the form of leaky translation of UUA codons in the absence of cognate tRNA. The leaky translation of AdpA_gh_ in a *ΔbldA_gh_* background provides some clues about the early stages of moenomycin biosynthesis as well as morphological differentiation in *S. ghanaensis*, when there would be little or no mature tRNA^Leu^_UAA_ in the cells [18,39]. Just a small amount of AdpA_gh_, available during early stages of growth in the absence of BldA_gh_, could be sufficient to activate transcription from *bldA_gh_p* leading to an avalanche-like increase in *bldA_gh_* expression. Once available, charged tRNA^Leu^_UAA_ could then amplify the translation of *adpA_gh_* and other, as-yet-unknown, UUA^+^ genes that lead to the downregulation of *adpA* and *moeE5* promoters. Our data confirm the presence of a regulatory feedback loop that amplifies a signal in dual regulation of BldA–AdpA in *S. ghanaensis*, as was previously shown in *S. griseus* [39].

The increased transcription of *adpA_gh_* from constitutive promoter *ermE* improved moenomycin production 2.5-fold in spite of the fact that (as our work shows) it is the translation efficiency of UUA-containing *adpA_gh_* mRNA that should determine the degree of activation of *moe* genes. At the moment, we cannot fully explain our results although several possible scenarios can be outlined. First, once the charged tRNA^Leu^_UAA_ is available, it might eventually lead to increased moenomycin production in the cells overexpressing *adpA_gh_* compared with the wild-type cells (note that we determine moenomycin production in the one time-point, which represents the total moenomycin produced over 72 h of growth). Second, if *adpA_gh_* mRNA is increased it might increase the probability of its mistranslation; this may also trigger moenomycin overproduction. Whatever the real mechanism is, it is practically useful because antibiotic titre improvement is a key requirement for the industry and it was one of the motivations for this work. In the case of CSR-free gene clusters, random mutagenesis and screening remain the only practical means to improve secondary metabolite production [59]. Recombinant DNA technology has yet to prove its utility for many industrial needs. Here, we demonstrate that the regulatory network *bldA–adpA–absB* is a cross-organism and large-effect system that can be harnessed to generate improved moenomycin producers. Upon combining *absB_gh_* deletion and *adpA_gh_* overexpression in *S. ghanaensis*, we observe, on average, a sevenfold increase in moenomycin production (data not shown). We anticipate that moenomycin titres can be further improved by bypassing *bldA_gh_* regulation, through the elimination of TTA codons from *moe* genes and *adpA_gh_*. Hence, genetic manipulations of the genes studied here could be a component of rational improvement of moenomycin producers. Recent studies [60,61] and several lines of evidence discussed above point to the fact that regulatory effects of *adpA* and *bldA* on SM are widespread and this could be exploited in other biosynthetic pathways. The amenability of SM to rational manipulations is also highlighted by a recent genome-wide study of the clavulanic acid overproducer, in which it was found that a small number of genetic changes, including AdpA overexpression, appeared to be associated with the desired phenotype [62].

## 5. Material and methods

### 5.1. Bacterial strains, plasmids and culture conditions

Strains and plasmids used in this study are described in the electronic supplementary material, table S2. *Escherichia coli* strains were grown in Luria-Bertani medium. *Streptomyces* strains were grown on SM and oatmeal agar media and in TSB and R2YE liquid media. Unless otherwise stated, *S. ghanaensis* was grown at 37°C and other streptomycetes at 30°C, with shaking at 200 r.p.m. All constructs were transferred into *Streptomyces* conjugally. The presence and stability of inheritance of *φ*C31-based constructs in streptomycetes was checked as described earlier [63,64].

### 5.2. Procedures for DNA manipulation

Oligonucleotides used in this work are listed in the electronic supplementary material, table S3. Standard procedures were used for plasmid/chromosomal DNA isolation, subcloning and analysis [65]. Polymerase chain reactions (PCRs) were performed using recombinant Pfu DNA polymerase (Fermentas) and all PCR products were sequenced. RedET-mediated gene replacements in cosmids and plasmids were carried out with the help of REDIRECT system [66]. All constructs were verified by sequencing, PCR or restriction mapping.

### 5.3. Quantitative analysis of moenomycins production

Growth of the strains, moenomycin extraction, conditions of LC-MS and quantitative analysis of the data are described by Ostash *et al*. [2] and Makitrynskyy *et al*. [10]. The levels of moenomycin production were calculated from at least three independent experiments and referred back to equal amounts of dry biomass (10 mg) in different strains. The cells were exhaustively extracted three times; the fourth extraction did not contain any measurable amounts of moenomycins confirming that all moenomycin had already been recovered (data not shown). The following compounds were monitored via LC/MS in *S. ghanaensis* extracts: MmA ([M-H]^−^ = 1580.6 Da) and nosokomycin B (NoB; [M-H]^−^ = 1484.6 Da). The mixture of these two equidominant compounds [64] is referred to as moenomycin in this work. Cosmid moeno38-5 directs the biosynthesis of nosokomycin B_1_ (NoB_1_; [M-H]^−^ = 1500.6 Da) and its production was followed in the extracts of heterologous hosts (*S. lividans* and *S. coelicolor*). LC/MS data were acquired on Agilent 1110 LC/MSD and Bruker Esquire 3000 ESI-MS spectrometers.

### 5.4. Identification of AdpA_gh_-binding sites

To identify conserved AdpA-binding sites (AdpAbs) in *S. ghanaensis*, known AdpAbs sequences were collected from GenBank. This dataset was used as input for the MEME software tool [67] to search for the consensus motif. Search for the occurrence of the identified motif within *moe* clusters, *bldA_gh_* and *adpA_gh_* promoter regions was performed using FIMO software suite [68].

### 5.5. Semiquantitative RT-PCR

Mycelia of *S. ghanaensis* were harvested in moenomycin production phase (72 h) and processed as described previously [10].

### 5.6. Construction of the *Streptomyces ghanaensis* *ΔabsB_gh_* and plasmids for complementation experiments

A construct for *absB*_*gh*_ knockout was prepared as follows. A 2.5 kb DNA fragment containing *absB_gh_* and its flanking regions were amplified from *S. ghanaensis* genomic DNA by PCR using primers absBgh_kn_for and absBgh_kn_rev. The PCR product was ligated to SmaI-digested pBluescriptKS+ to yield pBlabsBgh-kn. The *loxP* site-flanked apramycin resistance cassette (*aac(3)IV*) from plasmid pLERECJ was amplified with primers red_absBgh_kn_for and red_absBgh_kn_rev. The resulting amplicon was used to replace the coding sequence of *absB_gh_* in pBlabsBgh-kn via recombineering, giving pBlabsBgh-kn::aac(3)IV. The latter was digested with BamHI and EcoRI and the fragment containing the *absB_gh_::aac(3)IV* mutant allele was cloned into the same sites of pKC1139Km to yield pKCabsB-kn::aac3(IV). *Streptomyces ghanaensis* transconjugants carrying the latter were selected for resistance to apramycin (25 μg ml^−1^). To generate *S. ghanaensis* single-crossover Am^r^Km^r^ mutants, initial transconjugants were incubated at 40°C for 5 days, and then screened for apramycin resistance and kanamycin sensitivity (an indicative of vector loss and double crossover). Replacement of *absB_gh_* with *aac(3)IV* in *S. ghanaensis* *ΔabsB_gh_::aac(3)IV* was confirmed by PCR (primers absBgh_ex_for and absBgh_ex_rev; data not shown). The Cre-expressing helper plasmid pUWLCre was then introduced into *S. ghanaensis* *ΔabsB_gh_::aac(3)IV* to evict *aac(3)IV* from its genome. The pUWLCre^+^ transconjugants resistant to tiostrepton were incubated on oatmeal agar plates and selected for apramycin sensitivity. The helper plasmid was lost after two subsequent passages of selected Am^s^ clone in the absence of thiostrepton. Excision of *aac(3)IV* from the *S. ghanaensis* *ΔabsB_gh_* genome was confirmed by PCR (primers absBgh_ex_for and absBgh_ex_rev; data not shown).

A set of plasmids containing *absB_gh_* gene along with its upstream region of different lengths (figure 1) was constructed for complementation analysis. To create a plasmid pSOKabsBgh-exp, a 1.1 kb fragment carrying entire *absB_gh_* with its 150 bp 5′-region was amplified from *S. ghanaensis* genomic DNA using primers absBgh_ex_for and absBgh_ex_rev. The obtained amplicon was cloned into integrative VWB-based vector pSOK804 digested with EcoRV to give pSOKabsBgh-exp.

To construct plasmid pSOKEabsBgh-exp, where transcription of *absB_gh_* is under *ermEp* control, the above 1.1 kb PCR fragment was first cloned into EcoRV-treated pKC1218E, yielding pKCEabsBgh-exp. Then pKCEabsBgh-exp was digested with HindIII and EcoRI and 1.4 kb DNA fragment harbouring *absB_gh_* plus *ermEp* was ligated to pSOK804, digested with respective endonucleases, to generate pSOKEabsBgh-exp.

To create a plasmid pSOKabsBgh-II encompassing two genes, *SSFG_02130.1* and *SSFG_02129.1* (*absB_gh_*), along with the 200 bp upstream region, a 1.4 kb DNA fragment was amplified using primers absB-gh-II-for and absB-gh-II-rev. The resulting amplicon was cloned into EcoRV-treated pSOK804 to give pSOKabsBgh-II.

Plasmid pSOKabsBgh-III is based on pSOK804 and carries a 2.2 kb DNA fragment containing three genes, *SSFG_02131.1*, *SSFG_02130.1* and *SSFG_02129.1* (*absB_gh_*), along with the 250 bp upstream region. It was constructed by cloning an amplicon generated with primers absB-gh-IІI-for and absB-gh-IІI-rev into EcoRV site of pSOK804.

### 5.7. Construction of the *Streptomyces ghanaensis* *ΔadpA_gh_* and plasmid for complementation experiment

A 3.5 kb DNA fragment containing *adpA_gh_* and its flanking regions was amplified from the chromosome of *S. ghanaensis* using primers adpA_kn_for and adpA_kn_rev. The resulting amplicon was ligated to EcoRV-digested pBluescriptKS+ to yield pBladpAkn. To replace *adpA_gh_* the *aac(3)IV* cassette from pLERECJ was amplified using primers adpA_red_for and adpA_red_rev, and the resulting amplicon was used for recombineering-mediated replacement of *adpA_gh_* within pBladpAkn to give pBladpA-kn::aac(3)IV. The latter was further used as a template in PCR for amplification (primers adpA_kn_for and adpA_kn_rev) of a 3.4 kb DNA fragment harbouring *ΔadpA_gh_::aac(3)IV*. The obtained amplicon was cloned into EcoRV-digested vector pKC0702. The final *adpA_gh_* knockout plasmid was labelled pKCHadpA-kn::aac(3)IV. Generation of *ΔadpA_gh_* mutant was carried out as described above. Mutant phenotype of *S. ghanaensis* *ΔadpA_gh_::aac(3)IV* was confirmed by PCR using primers adpA_exp_for and adpA_exp_rev. Generation and verification of *aac(3)IV*-evicted strain *ΔadpA_gh_* was carried out as described for *ΔabsB_gh_* strain (primers adpA_for and adpA_rev; data not shown).

For the complementation of *S. ghanaensis* *ΔadpA_gh_*, a 1.9 kb fragment carrying *adpA_gh_* with its promoter region was amplified with primers adpA_for and adpA_rev_compl. The resulting amplicon was digested with XbaI and EcoRV and cloned into respective sites of pSET152, to give pSETadpA-exp.

For *adpA_gh_* expression under *ermEp* control, a 1.4 kb fragment containing only the coding sequence of *adpA_gh_* was amplified with primers adpA_exp_for and adpA_exp_rev. The amplicon was digested with EcoRV and EcoRI and ligated to EcoRV–EcoRI-linearized pTES to generate pTESaadpa-exp.

### 5.8. Construction of the *ΔbldA_gh_* strain and plasmid for complementation experiment

The 2.0 kb *S. ghanaensis* genomic regions flanking *bldA_gh_* were amplified with primers bldA-left-up plus bldA-left-rp (‘left’ homology arm) and bldA-right-up plus bldA-right-rp (‘right’ arm). ‘Left’ and ‘right’ amplicons were digested with HindIII + XbaI and XbaI + EcoRI, respectively, and cloned into HindIII–EcoRI-digested pKC1139. The resulting *bldA_gh_* knockout plasmid pKC1139bldA-del contains markerless deletion of the 87 bp *bldA_gh_* coding sequence. Manipulations of pKC1139bldA-del^+^ transconjugants to generate the *bldA_gh_* knockout strain were essentially the same as described above, except that double crossover clones were screened among those displaying impaired sporulation, as no antibiotic selection was possible. Diagnostic PCR with primers bldAXbaIup and bldA-diagn-rp and sequencing confirmed the deletion of the 87 bp *bldA_gh_* sequence from the genome of *ΔbldA_gh_*. For complementation and expression experiments, the *bldA_gh_* coding region along with the 320 bp upstream segment was amplified with primers bldAXbaIup and bldAEcoRIrp and cloned into respective sites of pSET152 to yield pSET152bldA.

### 5.9. Construction of GusA reporter plasmids and β-glucuronidase activity measurements

To probe the activities of *moeE5*, *absB_gh_, adpA_gh_* and *bldA_gh_* promoters, DNA fragments containing putative promoter regions (500 bp upstream of the translation start codons) were amplified by PCR using upstream primers carrying an XbaI site and downstream primers carrying a KpnI site (primers moeE5_for and moeE5_script_rev for *moeE5p*; absB_for and absB_script_rev for *absB_gh_p*; adpA_for and adpA_script_rev for *adpA_gh_p*; bldA_for and bldA_script_rev for *bldA_gh_p*). The *moeE5p*, *absB_gh_p*, *adpA_gh_p* and *bldA_gh_p* fragments were cloned into XbaI–KpnI-digested pGUS, to give plasmids pmoeE5script, pabsBscript, padpAscript and pbldAscript, respectively.

To investigate the expression of *moeE5* and *adpA_gh_* on the translational level, DNA fragments containing the entire stop codon-free genes with putative promoter (500 bp upstream of the translation start codons) were amplified by PCR using upstream primers carrying XbaI site and downstream primers carrying an EcoRV site (primers moeE5_for and moeE5_rev for *moeE5*; adpA_for and adpA_rev for *adpA_gh_*). The *moeE5* and *adpA_gh_* fragments were cloned into XbaI–EcoRV-digested pGUSHL4aadA [36], an integrative *Streptomyces* vector where the examined gene is fused to the *gusA* reporter gene through the helical linker HL4 [69], yielding pmoeE5transl and padpAtransl, respectively. In control experiments, promoterless *moeE5* and *adpA_gh_* genes without stop codon were amplified by PCR using upstream primers carrying XbaI site and downstream primers carrying EcoRV site (primers moeE5_for_contr and moeE5_rev for *moeE5*; adpA_for_contr and adpA_rev for *adpA_gh_*) and cloned in XbaI–EcoRV-treated pGUSHL4aadA, giving pmoeE5contr and padpAcontr, respectively.

The spore suspensions (2 × 10^5^ cfu) of streptomycetes reporter plasmid-bearing strains were inoculated in 300 ml flasks with 100 ml of TSB, and grown for 30 h. One millilitre of the preculture was inoculated into fresh TSB medium (100 ml) and grown for 24–28 h (depending on experiment). Mycelium was harvested, washed twice with distilled water, then resuspended in lysis buffer (50 mM phosphate buffer (pH 7.0), 0.1% Triton X-100, 5 mM DTT, 4 mg ml^−1^ lysozyme) and incubated for 30 min at 37°C. Lysates were centrifuged for 10 min at 5000 r.p.m. Then, 0.5 ml of lysate was mixed with 0.5 ml of dilution buffer (50 mM phosphate buffer (pH 7.0), 5 mM DTT, 0.1% Triton X-100) supplemented with 5 µl 0.2 M *p-*nitrophenyl-β-d-glucuronide and used for measuring optical density at *λ* = 415 nm every minute during 20 min of incubation at 37°C. As a reference, a 1 : 1 mixture of lysate and dilution buffer was used.

### 5.10. Expression and purification of His-tagged AdpA_gh_

For the production of C-terminal hexahistidine-tagged AdpA_gh_, the coding region of gene *adpA_gh_* was amplified with primers AdpA_pr_for and AdpA_pr_rev from *S. ghanaensis* chromosomal DNA. PCR product was purified and cloned into NcoI–XhoI cloning sites of expression vector pET24b, giving pETAdpAgh.

*Escherichia coli* BL21-GOLD cells harbouring the pETAdpAgh were grown in YTB medium containing 50 μg ml^–1^ ampicillin and kanamycin until The OD_600_ reached 0.8–1.0. Expression of AdpA_gh_ was induced with 0.4 mM IPTG at 20°C for 16–18 h. After incubation, cells were harvested by centrifugation and lysed in wash buffer (50 mM sodium phosphate, 300 mM sodium chloride) by French press. As AdpA_gh_ was expressed in both soluble and insoluble fractions, the lysate without centrifugation was directly mixed with Cobalt affinity resin. Purification of the protein was performed according to TALON Metal Affinity Resin manual (Clontech). The resin with attached protein was loaded on a column, washed with PBS. AdpA_gh_ was eluted with PBS containing 150 mM imidazole. Aliquots were examined using SDS-polyacrylamide gel electrophoresis and Coomassie blue staining. The eluted fraction was washed from imidazole by dialysis with a storage buffer (50 mM disodium hydrogen phosphate, 300 mM sodium chloride, 1 mM EDTA, 25% glycerol, pH = 7). Purified AdpA_gh_ samples were stored at –20°C in storage buffer. Protein concentration was determined according to the Bio-Rad DC Protein Assay.

### 5.11. Gel electrophoretic mobility shift assay

The 500 bp promoter regions of the targeted genes (*adpA_gh_*, *bldA_gh_*, *moeE5*, *moeK5*, *moeO5*) were used in EMSA. These probes were amplified from chromosomal DNA of *S. ghanaensis* by PCR using primers adpA_for and adpA_script_rev for *adpA_gh_*; bldA_for and bldA_script_rev for *bldA_gh_*; moeE5_for and moeE5_script_rev for *moeE5*; moeK5_for and moeK5_script_rev for *moeK5*; moeO5_for and moeO5_script_rev for *moeO5* (see the electronic supplementary material, table S3). A total of 10 pmol of each probe was 5′-end labelled with 20 pmol [γ-32P] using T4 polynucleotide kinase according to established protocols (Fermentas). Unincorporated labelled dATP was removed using ProbeQuant G-50 Micro columns (GE Healthcare). A total of 20 fmol of labelled probe was incubated with purified 1.1, 4.4, 11, 22, 44, 88 pM His-tagged AdpA_gh_ at 25°C for 15 min in 15 µl binding buffer (20 mM Tris–HCl (pH 8.0), 1 mM EDTA, 1 mM DTT, 100 mM KCl, 10 mM MgCl_2_, 10% glycerol) containing 1 µg of poly(dI-dC). The reactions products (protein-bound and free DNA) were separated on 4% non-denaturing polyacrylamide gel in TBE-buffer. The gels were visualized by phosphorimaging.

### 5.12. Western blotting MoeE5

Plasmid and conditions for expression and purification of N-terminal thioredoxin/His6-tagged MoeE5 protein in *E. coli* were previously described [2]. Purified recombinant MoeE5 protein was used as antigen to raise antibodies in a rabbit (as performed by Jackson ImmunoResearch laboratories (West Grove, PA, USA)). The same batch of *S. ghanaensis* mycelia was used for RT-PCR and Western blot analysis. Briefly, biomass samples were taken from −80°C, thawed on ice and resuspended in small volume of PBS. The mixture was French-pressed three times, centrifuged and supernatant taken for further analysis. Twenty microgram protein samples were separated in 7.5% SDS-polyacrylamide gels and upon blotting were probed with a 1 : 1000 dilution of the primary antiserum.

## Supplementary Material

Supplementary tables, figure and references
