# Supplementary material for: Pleiotropic regulatory genes bldA, adpA and absB are implicated in production of phosphoglycolipid antibiotic moenomycin
Source: Open Biol. 2013 Oct;3(10):130121. doi: 10.1098/rsob.130121 (PMC3814723; doi:10.1098/rsob.130121)
Supplement: Supplementary tables, figure and references [file rsob130121supp1.pdf]

Electronic Supplementary Materials

to the paper of Makitrinskyy R. et al. Pleiotropic regulatory genes *bldA*, *adpA* and *absB* are implicated in production of phosphoglycolipid antibiotic moenomycin

Table and Figure Captions to ESM

Table S1. $AdpA_{gh}$ paralogs encoded by *S. ghanaensis* genome (BLAST searches)

Table S2. Strains and plasmids used in this study

Table S2 footnote. Am^r, apramycin resistant; Km^r , kanamycin resistant; Sp^r, spectinomycin resistant; Ap^r, ampicillin resistant; Hy^r , hygromycin resistant; Tsr^r, thiostrepton resistant.

Table S3. List of the primers used in this work

Figure S1. Typical total ions chromatograms showing the presence of moenomycin A (MmA) and nosokomycin B (NoB) in crude methanol extracts from wild type ATCC14672 strain (**a**), $\Delta absB_{gh}$ strain (**b**), and the absence of moenomycins in the extracts from $\Delta adpA_{gh}$ (**c**) and $\Delta bldA_{gh}$ (**d**). X-axis, retention time (min); Y-axis – intensity of mass peaks (arbitrary units). Exact separation (and, consequently, unambigious identification) of all compounds present in the crude extract was not possible under our experimental conditions; nevertheless, new mass-peak seemed to be present in $\Delta adpA_{gh}$ and $\Delta bldA_{gh}$ extracts (highlighted with green rectangle), where one peak is present in all but $\Delta adpA_{gh}$ extract (red rectangle).

Figure S2. Purification of the His-tagged $AdpA_{gh}$. Lanes: 1, flow through; 2, purified $AdpA_{gh}$; 3, prestained protein marker (New England Biolabs). Arrow indicates His-tagged $AdpA_{gh}$ as verified by Western blot (data not shown)

Figure S3. Competition assay between labeled and unlabeled $adpA_{gh}$ promoter regions. $AdpA_{gh}$ protein (23 pM) was present in all reactions shown on the gel. Assay and P^{32} labeling conditions – see the main text.

Figure S4. $AdpA_{gh}$ is unable to bind *sco3812* promoter region. Lane 1 – P^{32}-labeled *sco3812p* (20 fM); lane 2 – P^{32}-labeled *sco3812p* (20fM) plus $AdpA_{gh}$ protein (2.2 nM)

Table S1. AdpA$_{gh}$ paralogs encoded by *S. ghanaensis* genome (BLAST searches)

**Name**	**Size, aa**	**ID/SI***	**e-value**	**Gaps**
SSFG_02454	329	49/62	$7e^{-108}$	2/324
SSFG_04078	346	46/56	$2e^{-96}$	6/321
SSFG_00572	322	45/57	$5e^{-93}$	7/323
SSFG_00931	320	39/54	$1e^{-91}$	2/319
SSFG_03135	334	46/57	$6e^{-91}$	1/309
SSFG_06690	318	43/55	$7e^{-89}$	6/321

* Identity (ID) and similarity (SI) to AdpA$_{gh}$, %

Table S2. Strains and plasmids used in this study.

Strain/plasmid	Description/function	Source
Strains		
S. ghanaensis ATCC14672	Wild type (WT) moenomycin producer	ATCC
S. ghanaensis $\Delta adpA_{gh}$	WT derivative, $\Delta adpA_{gh}$ deletion	This study
S. ghanaensis $\Delta bldA_{gh}$	WT derivative, $\Delta bldA_{gh}$ deletion	This study
S. ghanaensis $\Delta absB_{gh}$	WT derivative, $\Delta absB_{gh}$ deletion	This study
S. coelicolor M145	Wild type, $SCP1^-$, $SCP2^-$	Kieser *et al*. (2000)
S. coelicolor J3410	M145 derivative, *Δrnc* deletion mutant	Sello and Buttner, (2008)
S. coelicolor M851	M145 derivative, *ΔbldH* deletion mutant	Takano *et al*. (2003)
S. lividans 1326	Wild type strain	Kieser *et al*. (2000)
S. lividans J1725	1326 derivative, *bldA* inactivation	Leskiw *et al.* (1991)
E. coli DH5α	Host strain for DNA cloning	Hanahan, (1985)
E. coli ET12567	Host for DNA conjugation	Kieser *et al*. (2000)
E. coli BW25113	Host for DNA recombineering	Gust *et al*. (2004)
E. coli BL21-GOLD	Host for $AdpA_{gh}$ protein expression	Stratagene
B. cereus ATCC19637	Moenomycin-sensitive test-culture	ATCC
Cosmid		
moeno38-5	SuperCos1-based, carries entire *moe* cluster 1 except *moeA4*, *moeB4*, *moeR5*, *moeS5*; $int^{\varphi C31}$,$oriT^{RK2}$, Hy^rKm^r	Makitrynskyy *et al*. (2010)
Plasmids		
pBluescriptIIKS+	General purpose cloning vector, Ap^r	MBI Fermentas
pLERECJ	Carrying *aac(3)IV* flanked by *loxP*-sites, Am^rAp^r	A. Luzhetskyy
pUWLCre	Carrying *cre* under *ermEp*, Tsr^r	Fedoryshyn *et al.* (2008)
pSOK804	VWB-based integrative vector, Am^r	Sekurova *et al.* (2004)
pKC1139	*E. coli*/*Streptomyces* shuttle vector with temperature sensitive replicon pSG5, Am^r	Muth *et al*. (1989)
pKC1139Km	pKC1139 carrying *kan* instead of *aac(3)IV*, Km^r	M. Myronovskyi, pers. comm.
pKC1218E	pKC1218 carrying *ermEp*, Am^r	Ostash *et al*. (2004)
pKC0702	pKC1139 carrying *hph* instead of *aac(3)IV*, Hy^r	A. Luzhetskyy
pSET152	φC31-based integrative vector, Am^r	Bierman *et al*. (1992)
pTES	pSET152 carrying *ermEp*; Am^r	Hermann *et al.* (2012)
pGUS	Promoter probe vector; pSET152 carrying reporter gene *gusA*, Am^rSp^r	Myronovskyi *et al.* (2011)
pET24b	Vector for protein purification; Km^R	Novagen
pGUSHL4aadA	pTES-derivative for translational fusion experiments, Am^rSp^r	Myronovskyi *et al.* (2011)
pUZ8002	Expression of proteins for plasmid conjugation *in trans* , Km^r	Kieser *et al*. (2000)
pIJ790	Expression of proteins Bet, Exo and Gam for recombineering, Cm^r	Gust (2009)
pBlabsBgh-kn	pBluescriptIIKS+ carrying a 2.5 kb DNA fragment containing $absB_{gh}$, Ap^r	This study
pBlabsBgh-kn::aac(3)IV	pBlabsBgh-kn, $\Delta absB_{gh}$ deletion, Am^rAp^r	This study
pKCabsB-kn::aac(3)IV	pKC1139Km, $absB_{gh}$*::aac(3)IV*, Am^rKm^r	This study
pSOKabsBgh-exp	pSOK804 carrying $absB_{gh}$, Am^r	This study
pKCEabsBgh-exp	pKC1218E, carrying $absB_{gh}$, Am^r	This study
pSOKEabsBgh-exp	pSOK804, *ermEp*-$absB_{gh}$ fusion, Am^r	This study
pSOKabsBgh-II	pSOK804 carrying *SSFG_02130.1* and	This study

	SSFG_02129.1 (*absB*$_{gh}$), Am^r	
pSOKabsBgh-III	pSOK804 carrying *SSFG_02131.1*, *SSFG_02130.1*, *SSFG_02129.1* (*absB*$_{gh}$), Am^r	This study
pBladpAkn	pBluescriptIIKS+ carrying a 3.5 kb DNA fragment containing *adpA*$_{gh}$	This study
pBladpA-kn::aac(3)IV	pBladpAkn, *ΔabsB*$_{gh}$ deletion, Am^{r}Ap^r	This study
pKCHadpA-kn::aac(3)IV	pKC0702, *adpA*$_{gh}$*::aac(3)IV*, Am^{r}Hy^r	This study
pSETadpA-exp	pSET152 carrying *adpA*$_{gh}$ gene, Am^r	This study
pTESaadpA-exp	pTES, *ermEp-adpA*$_{gh}$ fusion, Am^r	This study
pmoeE5script	pGUS, *moeE5p-gusA* fusion, Am^{r}Sp^r	This study
pmoeO5script	pGUS, *moeO5p-gusA* fusion, Am^{r}Sp^r	This study
pabsBscript	pGUS, *absB*$_{gh}$*p-gusA* fusion, Am^{r}Sp^r	This study
padpAscript	pGUS, *adpA*$_{gh}$*p-gusA* fusion, Am^{r}Sp^r	This study
pbldAscript	pGUS, *bldA*$_{gh}$*p-gusA* fusion, Am^{r}Sp^r	This study
pmoeE5transl	pGUSHL4aadA, *moeE5-gusA* fusion, Am^{r}Sp^r	This study
pmoeE5contr	pGUSHL4aadA, promoterless *moeE5-gusA* fusion, Am^{r}Sp^r	This study
padpAtransl	pGUSHL4aadA, *adpA*$_{gh}$*-gusA* fusion, Am^{r}Sp^r	This study
padpAcontr	pGUSHL4aadA, promoterless *adpA*$_{gh}$*-gusA* fusion, Am^{r}Sp^r	This study
pET24bAdpA	pET24b carrying His$_6$-tagged AdpA$_{gh}$	This study
pSETbldA	pSET152 carrying *bldA*$_{gh}$ gene along with its 320 bp upstream region, Am^r	This study
pKC1139bldA-del	pKC1139-based *bldA*$_{gh}$ knockout construct, Am^r	This study

Table S3. List of the primers used in this work

Primer	Sequence
absBgh_kn_for	AAGTAGCCCGACTCGCCGTT
absBgh_kn_rev	CAACCCCCTTGTGCTCGACA
red_absBgh_kn_for	GTGAGAGGCACTGTGTCCACTGCCAAGAAGGCGGAAGACGCCGATATCTCTAGATACCG
red_absBgh_kn_rev	TCAGGCGGAGGCCGACGGGGCGTCGGCGCTCTTGCCGGTGGCAACAAAAGCTGGAGCTC
absBgh_ex_for	ATGGTTCCCCCTCCAGGGGC
absBgh_ex_rev	ACCGCCGGTCGCAGTGGAAG
absB-gh-II-for	GCACTGCCGATGCAGCCGGTGTGCCGG
absB-gh-II-rev	GCTCGGGCATGGTTCCCCCTCCAGGG
absB-gh-III-for	CCCTCCAGGGGCGGTTGTACG
absB-gh-III-rev	CCGTCGGGGACCCGGATT
adpA_kn_for	TGGAGCGGGCCTCGTCCTGA
adpA_kn_rev	CCAGGGCACCTCGGTGAGGT
adpA_red_for	CCGAGGAGCCGCGACCACCGAGGGGGGCTTAGCAGGATGGATATCTCTAGATACCG
adpA_red_rev	TCCGGCCCCGTCCGGTGTGCGCCGGTGCCGTCTACGTCAAACAAAAGCTGGAGCTC
adpA_exp_for	AAAATGCATACAACCGAGGAGCCGCGACCA
adpA_exp_rev	AAAGAATTCGCCTCCGGCCCCGTCCGGTGT
adpA_for	AAATCTAGAGCCGGCCGCGCGGCGGACCGTG
adpA_rev_compl	AAAGATATCGCCTCCGGCCCCGTCCGGTGT
moeE5_for	AAATCTAGAAGGGCGCCACCAGCTGGAGC
moeE5_script_rev	AAAGGTACCGATCAAGGCGGTGCATTGCG
moeE5_rev	AAAGATATCCAGCCGCGGCACGGACACCGA
moeE5_for_contr	AAATCTAGAAGCCAGTTGTGAGCGAAACAAGG
absB_for	AAATCTAGATCCAGCGACGCCGACTACCT
absB_script_rev	AAAGGTACCGAAGAGCCAGAATACTGGAC
adpA_script_rev	AAAGGTACCCCTCGGTTGTGTCGCTCCTG
adpA_rev	AAAGATATCCGGCGCGCTGCGCTGTCCCGGGAC
adpA_for_contr	AAATCTAGAAGCCGCGACCACCGAGGGGGGCT
bldA_for	AAATCTAGACTTCGACAGGTCCGTGGCGC
bldA_script_rev	AAAGGTACCAGGCCATGGGCTCCGCTTCG
AdpA_pr_for	AATGTTCGATACATATGAGCCACGACTCCACTGCCGC
AdpA_pr_rev	AATGTCTCGAGCGGCGCGCTGCGCTGTCCCG
moeK5_for	AAATCTAGACGCGCACCGACTGGCGTACC
moeK5_script_rev	AAAGGTACCTCTGGACCCGCGCTATCTGAC
moeO5_for	AAATCTAGAGCATAACCGCCGCACAAAGTC
moeO5_script_rev	AAAGGTACCCGTCCGGCTTGTCGTCCGGTC
bldA-left-up	AAAAGCTTGGTGGTCTCCACGACGTCG
bldA-left-rp	AAATCTAGAAGGCCATGGGCTCCGCTTCG
bldA-right-up	AAATCTAGACTGCGCCTACGGTGAGGCGCAC
bldA-right-rp	AAAGAATTCAGGACGAGCAGCCCGAGCGTG
bldAXbaIup	AAATCTAGATGCAGTCAACCGCTGCTCCGGTTC
bldAEcoRIrp	AAAGAATTCTGGTGCCCGGAGCCGGACTTG
bldA-diagn-rp	GTGCGCCTCACCGTAGGCGCAG

Figure S1

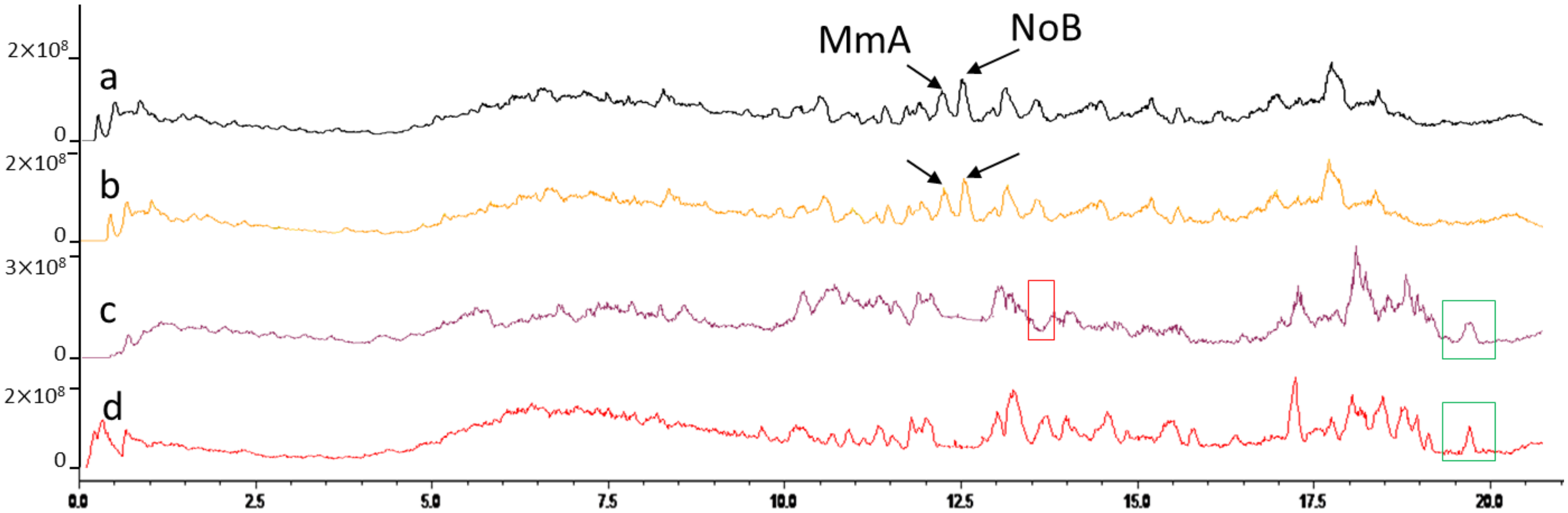

MmA
NoB
2×10⁸
a
0
2×10⁸
b
0
3×10⁸
c
0
2×10⁸
d
0
0.0
2.5
5.0
7.5
10.0
12.5
15.0
17.5
20.0

Figure S2

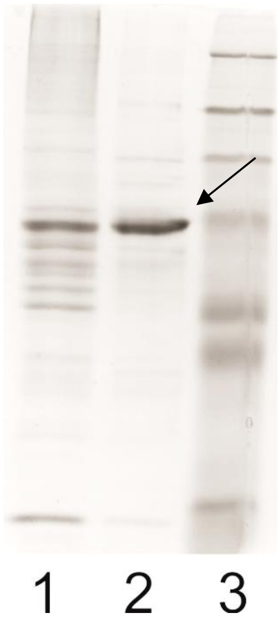

Figure S3

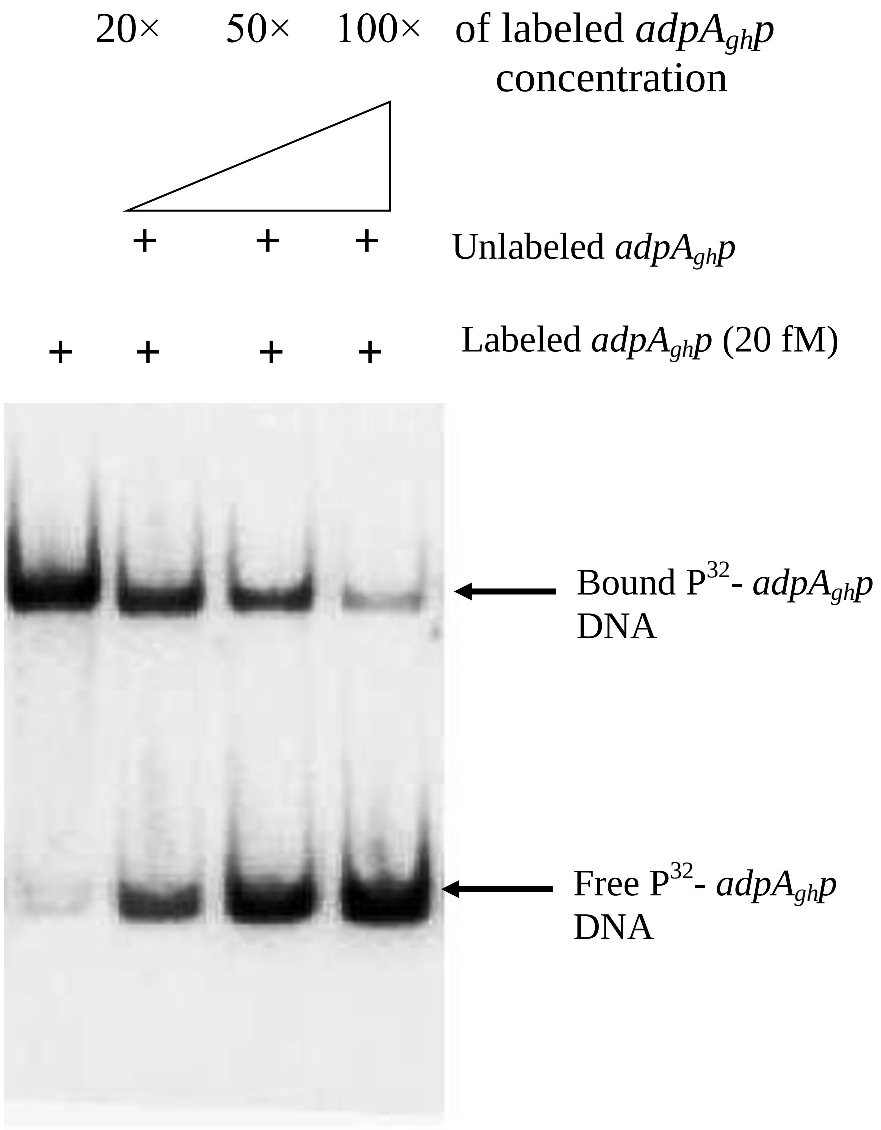
20×
50×
100×
of labeled *adpA*$_{gh}$*p* concentration
+ + + Unlabeled *adpA*$_{gh}$*p*
+ + + + Labeled *adpA*$_{gh}$*p* (20 fM)
Bound P^{32}- *adpA*$_{gh}$*p* DNA
Free P^{32}- *adpA*$_{gh}$*p* DNA

Figure S4

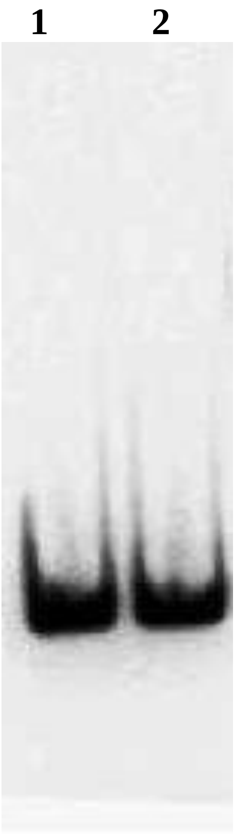

Supplementary references

Bierman, M., Logan, R., O'Brein, K., Seno, E., Rao, R., and Schoner, B. (1992) Plasmid cloning vectors for the conjugal transfer of DNA from *Escherichia coli* to *Streptomyces* spp. *Gene* **116:** 43–49.

Fedoryshyn, M., Welle, E., Bechthold, A., and Luzhetskyy, A. (2008) Functional expression of the Cre recombinase in actinomycetes. *Appl Microbiol Biotechnol* **78**: 1065–1070.

Gust, B., Chandra, G., Jakimowicz, D., Yuqing, T., Bruton, C.J., and Chater K.F. (2004) Lambda red-mediated genetic manipulation of antibiotic-producing *Streptomyces*. *Adv Appl Microbiol* **54:** 107–128.

Gust, B. (2009) Cloning and analysis of natural product pathways. *Methods Enzymol* **458:** 159–180.

Hanahan D. (1985) Techniques for transformation of *Escherichia coli*. In DNA Cloning. Edited by D.M. Glover. Oxford, UK: IRL Press 109–135.

Herrmann, S., Siegl, T., Luzhetska, M., Petzke, L., Jilg, C., Welle, E. (2012) Site-specific recombination strategies for engineering actinomycete genomes. *Appl Environ Microbiol* **78:** 1804–1812.

Kieser, T., Bibb, M.J., Buttner, M.J., Chater, K.F., and Hopwood, D.A. (2000) *Practical Streptomyces Genetics*. Norwich: John Innes Foundation.

Leskiw, B.K., Lawlor, E.J., Fernandez-Abalos, J.M., and Chater, K.F. (1991) TTA codons in some genes prevent their expression in a class of developmental, antibiotic-negative, *Streptomyces* mutants. *Proc Natl Acad Sci USA* **88:** 2461–2465.

Makitrynskyy, R., Rebets, Y., Ostash, B., Zaburannyi, N., Rabyk, M., Walker, S., and Fedorenko, V. (2010) Genetic factors that influence moenomycin production in streptomycetes. *J Ind Microbiol Biotechnol* **37:** 559–566.

Myronovskyi, M., Welle, E., Fedorenko, V., and Luzhetskyy, A. (2011) Beta-glucuronidase as a sensitive and versatile reporter in actinomycetes. *Appl Environ Microbiol* **77:** 5370–5383.

Muth, G., Nussbaumer, B., Wohlleben, W., and Pühler, A. (1989) A vector system with temperature-sensitive replication for gene disruption and mutational cloning in streptomycetes. *Mol Gen Genet* **6:** 1–8.

Ostash, B., Rix, U., Rix, L.L., Liu, T., Lombo, F., Luzhetskyy, A., *et al*. (2004) Generation of new landomycins by combinatorial biosynthetic manipulation of the *lndGT4* gene of the landomycin E cluster in *S. globisporus*. *Chem Biol* **11:** 547–555.

Sekurova, O.N., Brautaset, T., Sletta, H., Borgos, S.E., Jakobsen, M.O., Ellingsen T.E., *et al*. (2004) *In vivo* analysis of the regulatory genes in the nystatin biosynthetic gene cluster of *Streptomyces*

noursei ATCC11455 reveals their differential control over antibiotic biosynthesis. *J Bacteriol* **186:** 1345–1354.
Sello, J.K., and Buttner, M.J. (2008) The gene encoding RNase III in *Streptomyces coelicolor* is transcribed during exponential phase and is required for antibiotic production and for proper sporulation. *J Bacteriol* **190:** 4079–4083.
Takano, E., Tao, M., Long, F., Bibb, M.J., Wang, L., Li, W., *et al*. (2003) A rare leucine codon in *adpA* is implicated in the morphological defect of *bldA* mutants of *Streptomyces coelicolor*. *Mol Microbiol* **50:** 475–486.
